# Therapeutic Effects of *Dipterocarpus tuberculatus* with High Antioxidative Activity Against UV-Induced Photoaging of NHDF Cells and Nude Mice

**DOI:** 10.3390/antiox10050791

**Published:** 2021-05-17

**Authors:** Su Jin Lee, Ji Eun Kim, Yun Ju Choi, Jeong Eun Gong, So Hae Park, Bounleuane Douangdeuane, Onevilay Souliya, Ju Min Park, Hee Seob Lee, Bae-Hwan Kim, Dae Youn Hwang

**Affiliations:** 1 Department of Biomaterials Science (BK21 FOUR Program), College of Natural Resources and Life Science/Longevity & Wellbeing Research Center, Pusan National University, Miryang 50463, Korea; nuit4510@naver.com (S.J.L.); prettyjiunx@naver.com (J.E.K.); poiu335@naver.com (Y.J.C.); jegog@naver.com (J.E.G.); sohaehw@pusan.ac.kr (S.H.P.); 2 Life and Industry Convergence Research Institute/Laboratory Animal Center, Pusan National University, Miryang 50463, Korea; 3 Institute of Traditional Medicine, Ministry of Health, Vientiane 0103, Lao PDR; bounleuane.dd@gmail.com (B.D.); onevilay@gmail.com (O.S.); 4 Department of Food Science and Nutrition, College of Human Ecology, Pusan National University, Busan 46241, Korea; mminv8@naver.com (J.M.P.); heeseoblee@pusan.ac.kr (H.S.L.); 5 Department of Public Health, Keimyung University, Daegu 42601, Korea; kim9399@kmu.ac.kr

**Keywords:** *Dipterocarpus tuberculatus*, UV, photoaging, antioxidants, skin aging

## Abstract

To investigate the therapeutic effects of methanol extracts of *Dipterocarpus tuberculatus* Roxb. (MED) against UV-induced photoaging, we assessed for alterations in the antioxidant activity, anti-apoptotic effects, ECM modulation, skin appearances, and anti-inflammatory response in normal human dermal fibroblast (NHDF) cells and nude mice orally treated with MED. High levels of tannin content and high free radical scavenging activity to DPPH were determined in MED, while seven active components, namely, gallic acid, bergenin, ellagic acid, ε-viniferin, asiatic acid, oleanolic acid, and 2α-hydroxyursolic acid, were identified using LC–MS analyses. UV-induced alterations in the NO concentration, SOD activity, and Nrf2 expression were remarkably recovered in MED-treated NHDF cells. Moreover, the decreased number of apoptotic cells and G2/M phase arrest were observed in the UV + MED-treated groups. Similar recoveries were detected for β-galactosidase, MMP-2/9 expression, and intracellular elastase activity. Furthermore, MED treatment induced suppression of the COX-2-induced iNOS mediated pathway, expression of inflammatory cytokines, and inflammasome activation in UV-radiated NHDF cells. The anti-photoaging effects observed in NHDF cells were subsequently evaluated and validated in UV + MED-treated nude mice through skin phenotypes and histopathological structure analyses. Taken together, these results indicate that MED exerts therapeutic effects against UV-induced photoaging and has the potential for future development as a treatment for photoaging.

## 1. Introduction

Photoaging is the onset of chronological aging due to continuous long-term exposure to chronic UV radiation, including UVA and UVB. Photoaged skin is characterized by clinical phenotypes such as wrinkle formation, laxity, irregular pigment condensation, and thickening of the skin. These phenotypes are initiated by the photochemical generation of reactive oxygen species (ROS) in the skin connective tissue [1]. These ROS (such as peroxide anion, peroxide, and singlet oxygen) cause chemical modifications and oxidation of various cellular components including DNA, proteins, and lipids, subsequently resulting in oxidative stress [1,2]. During these processes, the activated MAP kinase signaling pathway promotes secretion of inflammatory cytokines, which induce the upregulation of MMP [3,4]. MMP mediates degradation of collagen and elastic fibers, thereby resulting in the formation of coarse wrinkles and sagging skin [5,6]. In terms of the onset mechanisms of photoaging, techniques to eliminate the oxidative stress are considered as important treatment strategies for photoaging.

The therapeutic effects of some natural products with antioxidative activity on UV-induced photoaging have been evaluated in various skin cells and animals, although the analyses factors were not constant in each study. The enhancement of MMP expressions and ROS production induced by UV irradiation were significantly inhibited by several natural products including *Gynura procumbens* Merr., *Polypodium leucotomos*, *Passiflora tarminiana*, and hawthorn [7,8,9,10]. UV- and ROS-induced DNA damage as well as antioxidant enzymes were also suppressed after treatment with *Polypodium leucotomos*, *Melissa officinalis*, hawthorn, or *Belamcandae rhizome* [8,10,11,12]. The above inhibitory effects were mediated by the MAP kinase signaling pathway, regulation of the Nrf2/ARE activator, or the NF-κB signaling pathway in skin fibroblasts and hairless mice [8,10,13]. In particular, anti-photoaging activity of most natural products is tightly linked to their antioxidant activity and antioxidants as a single component [8,9,10,11,12,14]. However, no study has elucidated the anti-photoaging effect and the mechanism of action of *Dipterocarpus tuberculatus* in skin fibroblasts, although a study on the possibility of anti-inflammatory effects of *D. tuberculatus* ethanol extracts has been reported [15].

The current study was undertaken to identify a novel natural product with high antioxidative activity for photoaging. The therapeutic effects and the mechanism of a methanol extract of *D. tuberculatus* stem (MED) was investigated in UV-irradiated skin fibroblasts and nude mice.

## 2. Materials and Methods

### 2.1. Preparation and Deposition of MED

The sample of MED (FBM 213-075) was kindly provided from the International Biological Material Research Center of the Korea Research Institutes of Bioscience and Biotechnology (Daejeon, Republic of Korea). Firstly, powder of *D. tuberculatus* Roxb. stem was mixed with methanol solution in a 1:10 ratio. The mixture was repetitively subjected to the three steps: (1) sonication for 15 min followed by incubation for 2 h, (2) 10 times per day for 3 days, and (3) filtered through a filter with 0.4 µm pore size. Then, this filtered solution was concentrated and lyophilized using a rotary evaporator (*n* = 1000 SWD, EYELA, Bohemia, NY, USA) and a speed vacuum concentrator (Modulspin 40, Biotron Co., Marysville, WA, USA). Finally, the prepared MED sample was dissolved in dimethyl sulfoxide (DMSO; Duchefa Biochemie, Haarlem, The Netherlands) to treatment concentrations.

### 2.2. Free Radical Scavenging Activity of MED

The scavenging activity of MED against 2,2-diphenyl-1-picrylhydrazyl (DPPH) radical was determined as a method with some modification described in a previous study [16]. Briefly, 12 different concentrations (1 to 2000 µg/mL) of MED were mixed with 100 µL 0.1 mM DPPH (Sigma-Aldrich Co., St. Louis, MO, USA) in a 95% ethanol solution. After the 30 min of incubation at room temperature, the absorbance of reaction mixture was determined using Versa Max plate reader (Molecular Devices, Sunnyvale, CA, USA) at 517 nm. The final data were represented as the reduction percent in absorbance, relative to the control. The IC_50_ value was defined the MED concentration that exerts a 50% reduction in DPPH radical scavenging activity.

### 2.3. Determination of Phytochemical Composition in MED

The total phenolic content (TPC), total flavonoid content (TFC), and total condensed tannin content (TCT) of MED were determined using the method with some modification, as previously described [17,18,19]. Briefly, the MED sample was reacted with appropriate reagent for each phytochemical component at a specific temperature. The absorbance of this mixture was determined at 765 or 500 nm using a Versa Max plate reader (Molecular Devices). Finally, the concentration of TPC, TFC, and TCT was presented as the gallic acid equivalent (mg), catechin equivalents (mg), and a purified (β)-catechin hydrate standard of the extract.

### 2.4. Liquid Chromatography–Mass Spectrometry (LC–MS) Analysis

LC-MS analysis was performed with an Agilent 1290 Infinity HPLC system (Agilent Technologies, Waldbronn, Germany), coupled with a hybrid quadrupole time-of-flight (Q-TOF) mass spectrometer (6530, Agilent Technologies). LC-MS signals were detected on a mass spectrometer operating in the negative ionization mode. An ACQUITY UPLC BEH C18 Column (2.1 × 100 mm, 1.7 μm) (Waters, Milford, MA, USA) was applied to chromatographic separation under the following conditions: 0.3 mL/min in flow rate, 10 μL of injection volume, 0.1% formic acid–water of mobile phase A, and 100% acetonitrile of mobile phase B. For MS detection, the operating parameters were as follows: gas temperature, 300 °C; gas flow, 9 L/min; nebulizer pressure, 45 psig; sheath temperature, 350 °C; sheath gas flow, 11 L/min; VCap, 4000 V; fragmentor voltage, 175 V. All the acquisition and analysis of data were controlled by MassHunter software (version B. 0600, Agilent Technologies).

### 2.5. Cell Viabilities

NHDF cells that are established from the dermis of juvenile foreskin, or adult skin from different locations, were purchased from the ATCC (Manassas, VA, USA). They were cultured in a humidified 5% CO_2_ and 95% oxygen atmosphere at 37 °C in Dulbecco’s modified Eagle’s medium (DMEM; Wellgene, Gyeongsan, Korea) supplemented with 10% fetal bovine serum, 2 mM glutamine, 100 U/mL penicillin, and 100 μg/mL streptomycin.

The viabilities of NHDF cells were measured using the MTT (3-[4,5-dimethylthiazol-2-yl]-2,5 diphenyltetrazolium bromide) assay (Sigma-Aldrich Co.). When density of NHDF cell reached 70–80%, they were divided into the following six groups: UV + Vehicle (DMSO)-treated group, UV + 1.25% of cold-pressed perilla oil group (UV + CPO-treated group), low-concentration MED group (100 μg/mL; UV + LMED-treated group), medium-concentration MED group (200 μg/mL; UV + MMED-treated group), high-concentration MED group (400 μg/mL; UV + HMED-treated group), and non-irradiated group (No-treated group). CPO was used as the positive control group (Po-treated group) due to its high antioxidant activity. Cells were treated with MED or CPO immediately after UVB irradiation, which was performed with a TL 20W/12 RS SLV/25 UVB Broadband TL lamp (Philips, Amsterdam, The Netherlands). Radiation intensities (mW/cm^2^) of UVB were measured at 30 cm from a light source using a UVP UVXTM Digital Radiometer (Analytik Jena US LLC, Upland, CA, USA). After incubation for 24 h, culture supernatants were discarded, followed by addition of 200 μL fresh DMEM and 50 μL MTT solution (20 mg/mL in 1× PBS) to each well, and subsequent incubation at 37 °C for 4 h. Finally, the formazan precipitates were then dissolved in DMSO (Duchefa Biochemie), and absorbance of each well was measured at 570 nm using a Versa max plate reader (Molecular Devices).

### 2.6. Nitric Oxide (NO) Concentration

The level of nitrite, which is the stable reaction product generated from NO with molecular oxygen, was used as an indicator of NO production. Briefly, NHDF cells in each well were treated with 55 mJ of UV, and subsequently with MED or CPO for 24 h, after which the supernatants were collected. For skin tissue, homogenates were collected and kept at −80 °C until use. Duplicates of 100 μL supernatant of homogenate or culture media were added to 96-well plates and mixed with 100 μL modified Griess reagent (Invitrogen, California, USA). The absorbance of each well was measured at 540 nm using a Versa max plate reader (Molecular Devices). A standard curve with increasing concentrations of sodium nitrite was generated in parallel and used for quantification.

### 2.7. Apoptotic Cell Analysis

Distribution of apoptotic cells were analyzed using a Muse^TM^ Annexin V and Dead Cell Kit (Millipore Co., Billerica, MA, USA), on the basis of the manufacturer’s protocol. NHDF cells were harvested following irradiation with 55 mJ UV and subsequent treatment with MED or CPO for 24 h. Harvested NHDF cells were suspended in DMEM (3 × 10^4^ cells/mL), and 100 μL of this cell suspension (1 × 10^4^ cells/mL) was then incubated with Muse^TM^ Annexin V and Dead Cell Kit (Millipore Co.) reaction reagent for 20 min at room temperature. Finally, cells of the reaction mixture were analyzed using a Muse^TM^ Cell Analyzer (Millipore Co.), and data are presented as the number of live and apoptotic cells.

### 2.8. Cell Cycle Analysis

The distribution of cell population in each phage of cell cycle was determined using a Muse™ Cell Cycle Kit (Millipore Co.), according to the manufacturer’s instructions. Briefly, NHDF cells were treated with 55 mJ UV, and subsequently with MED or CPO for 24 h. After centrifugation, the harvested cells were fixed with 70% EtOH for 3 h at −20 °C and incubated with the 200 μL of Cell Cycle Reagent at 37 °C for 30 min. The number of cells in each phase was analyzed by a Muse^TM^ Cell Analyzer (Millipore Co.).

### 2.9. β-Galactosidase Staining

The degree of β-galactosidase staining in NHDF cells was measured using the β-Galactosidase Detection Kit (Cell Signaling Technology Linc., Danvers, MA, USA), on the basis of the manufacturer’s protocol. NHDF cells were treated with 55 mJ UV and subsequently with MED or CPO for 24 h to detect the alteration of β-galactosidase activity. After fixation in fixative solution (1 mL) for 10–15 min, the cells were incubated with β-galactosidase staining solution (1 mL) at 37 °C at least overnight in a dry incubator. Finally, cells stained with blue color were observed at 200× magnification under a microscope (Leica Microsystems, Wetzlar, Germany).

### 2.10. Superoxide Dismutase (SOD) Activity Analysis

SOD activity in NHDF cells and skin tissue was determined using a SOD assay kit (Dojindo Molecular Technologies Inc., Rockville, MD, USA). Briefly, NHDF cells in 100 μL 1× PBS were lysed by repetitive freezing and thawing, and skin tissues were homogenized in 400 μL of sucrose buffer. After collection of cell lysate and tissue homogenates, they were diluted to 1/1, 1/2, 1/22, 1/23, 1/24, 1/25, and 1/26 with 1× PBS solution in a 96-well plate. Total lysate and homogenate in each well were mixed with WST-1 working solution (200 μL) and enzyme working solution (20 μL), and subsequently these mixtures were incubated at 37 °C for 20 min. The absorbance of each well was measured at 450 nm using a spectrophotometer, and calculation of SOD activity was attained using the following equation:

SOD activity (inhibition rate %) = [(A blank 1 − A blank 3) − ( A sample − A blank 2)]/(A blank 1 − A blank 3) × 100(1)

where A blank 1, 2, and 3 indicate the absorbance of blanks 1, 2, and 3, respectively, and “A sample” is the sample absorbance.

### 2.11. Intracellular Elastase Inhibition Assay

The intracellular elastase inhibition assay of each extract was measured using a modified method of Cannell. To obtain cell lysate and tissue homogenates, we lysed NHDF cells in 0.1 M Triton-HCl buffer containing 0.1% Triton-X, and skin tissue was homogenated in 0.2 M Triton-HCl (pH 8.0) buffer containing 0.1% Triton-X. These lysates and homogenates were then centrifuged for 10 min at 12,000 rpm, and the supernatant was collected as the elastase containing solution. About 100 μL elastase-containing solution was subsequently mixed with 20 μL substrate, followed by incubation at 37 °C for 20 min. The mixture was then cooled for 5 min, and absorbance was measured at 410 nm. The elastase inhibition was calculated as below:

Inhibition rate (%) = [1 − (Absorbance sample/Absorbance control)] × 100(2)

### 2.12. Western Blot Analysis

Pro-Prep Protein Extraction Solution (Intron Biotechnology Inc., Seongnam, Korea) was used to obtain total proteins from NHDF cells and skin tissues, on the basis of the manufacturer’s protocol. After determination of the protein concentration using a SMARTTM Bicinchoninic Acid Protein Assay Kit (Thermo Fisher Scientific Inc., Wilmington, DE, USA), we separated total proteins by 4–20% SDS-PAGE (sodium dodecyl sulfate–polyacrylamide gel electrophoresis) for 2 h, followed by transfer to nitrocellulose membranes at 40 V for 2 h. They were independently incubated overnight at 4 °C with the specific primary antibodies (Supplementary Table S1). These membranes were then incubated with 1:2000 diluted horseradish peroxidase (HRP)-conjugated goat anti-rabbit IgG (Invitrogen) at room temperature for 1 h. Enzyme on blots were developed using the Amersham ECL Select Western Blotting detection reagent (GE Healthcare, Little Chalfont, UK). Finally, chemiluminescence signals for each band were detected under FluorChemi^®^FC2 (Alpha Innotech Co., San Leandro, CA, USA).

### 2.13. Quantitative Real-Time PCR (RT-qPCR) Analysis

Total RNAs were purified from NHDF cells and skin tissues using RNAzol (Tet-Test Inc., Friendswood, TX, USA). After determination of total RNA concentrations, synthesis of complementary DNA (cDNA) was performed using the Superscript II reverse transcriptase (Thermo Fisher Scientific Inc.), and qPCR was performed using the cDNA template (1 μL), 2 × Power SYBR Green (6 μL; Toyobo Life Science, Osaka, Japan), and specific primers (Supplementary Table S2). Cycle (40 reaction) of qPCR consists of the following three stages: denaturation at 95 °C for 15 s, annealing at 70 °C for 60 s, and extension at 70 °C for 60 s. Fluorescence intensities for each reaction were measured at the end of the extension phase of each cycle. Threshold values for above intensities were set manually, and reaction cycles wherein the PCR products exceeded these fluorescence intensity thresholds during the exponential phase were considered as threshold cycles (Ct). Expressions of TNF-α, IL-6, IL-1β, and NF-κB genes were quantified with respect to β-actin (the housekeeping gene) by comparing Ct values at constant fluorescence intensity, as described by Livak and Schmittgen.

### 2.14. Experimental Design for Animal Study

The Pusan National University (PNU)–Institutional Animal Care and Use Committee reviewed and approved the protocol for animal study (approval no. PNU-2020-2700). Mice were housed at the PNU-Laboratory Animal Resources Center (LARC) accredited by the Korean Food and Drug Administration (KFDA) (unit 000231) and the Association for Assessment and Accreditation of Laboratory Animal Care International (AAALAC International) (unit 001525). Female athymic nude mice (7 weeks old) were purchased from the Central Lab Animal Inc. (Seoul, Korea). Drinking water and a standard irradiated chow diet (Samtako BioKorea Co., Osan, Korea) were provided ad libitum to mice throughout the experimental period. All mice used this study were bred under specific pathogen-free conditions (SPF) (50 ± 10% RH/23 ± 2 °C) under a strict light/dark cycle.

Briefly, 7-week-old nude mice (female, *n* = 32) were assigned to either a non-irradiated group (No group, *n* = 8) or a UV irradiation group (*n* = 24). The UV irradiation group was further divided into the UV + vehicle-treated group (1 × PBS, UV + Vehicle group, *n* = 8), UV + low-concentration MED group (100 mg/kg, UV + LMED group, *n* = 8), or UV + high-concentration MED group (200 mg/kg, UV + HMED group, *n* = 8). Mice in each group were orally administrated with the same volume of 1 × PBS, 100 mg/kg, or 200 mg/kg of MED three times a week for 4 weeks, after being irradiated with UVB.

After determination of the 1 minimal erythema dose, skin photoaging was then induced by irradiation at 1 minimal erythema dose at once for three times per week (Monday, Wednesday, and Friday) in the first week. After that, the dose of UV radiation was gradually increased by 1 minimal erythema dose per week, from 1 to 4 minimal erythema doses, and treatment was imparted in a similar manner.

After treatment for 4 weeks, skin phenotypes in the dorsal skins of mice were measured, and then skin tissue samples were collected from mice sacrificed using CO_2_.

### 2.15. Evaluation of Wrinkle Formation

Wrinkle formation on the dorsal skin was determined using DETAX System II (MIXPAC) and Double-Stick Disc (3M Health Care, Neuss, Germany), as previously described. After final treatment of UV and MED, skin surface replicas were attained by applying liquid silicon rubber to dorsal skins, delivered using the DETAX System II. Wrinkle image on each replica was taken with a digital camera connected to the Leica EZ4HD (Leica Microsystems). The wrinkle score was determined on the basis of depths and numbers of wrinkles as described by Bissett et al. [20], where grade 0 = no wrinkle formation, grade 1 = some shallow wrinkles, grade 2 = obvious wrinkles, and grade 3 = several deep wrinkles.

### 2.16. Skin Phenotypes Analysis

Four skin phenotypes, namely, TEWL, erythema severities, hydration, and melanin severities, were assessed on dorsal skins of mice after anesthesia with Alfaxan (Jurox, Kansas, USA; i.p., 80 mg/kg body weight). Corneometer TM300 (Courage and Khazaka Electronics, Cologne, Germany) and the Corneometer CM825 (Courage and Khazaka Electronics, Cologne, Germany) were used to measure TEWLs and hydration. Mexameter MX18 (Courage and Khazaka Electronics, Cologne, Germany) were used to determine erythema and melanin indices. Each measurement was performed in duplicate at three dorsal skin sites per mouse.

### 2.17. DNA Fragmentation Assay

Total genomic DNA was extracted from the dorsal skin of mice using the Wizard Genomic DNA Purification kit (Promega, Wisconsin, USA). Briefly, skin tissues were homogenized in 600 μL of nucleic lysis solution, followed by addition of RNase solution and Protein Precipitation Solution. The DNA was precipitated by centrifugation at 15,000× *g* for 5 min, after which the concentration was measured by Nano Drop Spectrophotometer (Allsheng, Hangzhou, China). Equal concentrations of genomic DNA were loaded onto a 1.2% agarose gel and electrophoresed for 40 min at a constant voltage (100 V). DNA was subsequently visualized by UV illumination (E-Graph, Atto, NY, USA).

### 2.18. Histopathological Analysis

After collection of skin tissues from nude mice, all tissues were fixed in 10% neutral buffered formaldehyde (pH 6.8), dehydrated in an alcohol dilution series, trimmed with a sharp knife, and embedded in paraffin wax. Tissue sections with 4 µm were deparaffinized with xylene solution (DaeJung Chemicals, Siheung, Korea) and rehydrated using an alcohol dilution series (100 to 70%). After washing with distilled water, skin tissues were stained with hematoxylin and eosin (H&E; Sigma-Aldrich Co.), and histopathological changes were observed using the Leica Application Suite (Leica Microsystems).

The distribution of mast cells was detected in the toluidine blue stained skin tissue using a previously described method [21]. After staining with 0.25% toluidine blue (Sigma-Aldrich Co.), total numbers of mast cells per square millimeter were determined using the Leica Application Suite (Leica Microsystems).

### 2.19. Statistical Significance Analysis

Statistical analyses were performed using the SPSS release 10.10 for Windows (IBM SPSS, SPSS Inc., Chicago, IL, USA). The significance of intergroup differences was determined by one-way analysis of variance followed by Tukey’s post hoc test for multiple comparisons. Results data are presented as mean ± SD, and *p*-values < 0.05 are considered as statistically significant.

## 3. Results

### 3.1. Anti-Oxidative Activity, Phytochemical Composition, and Active Components of MED

**Figure 1:**
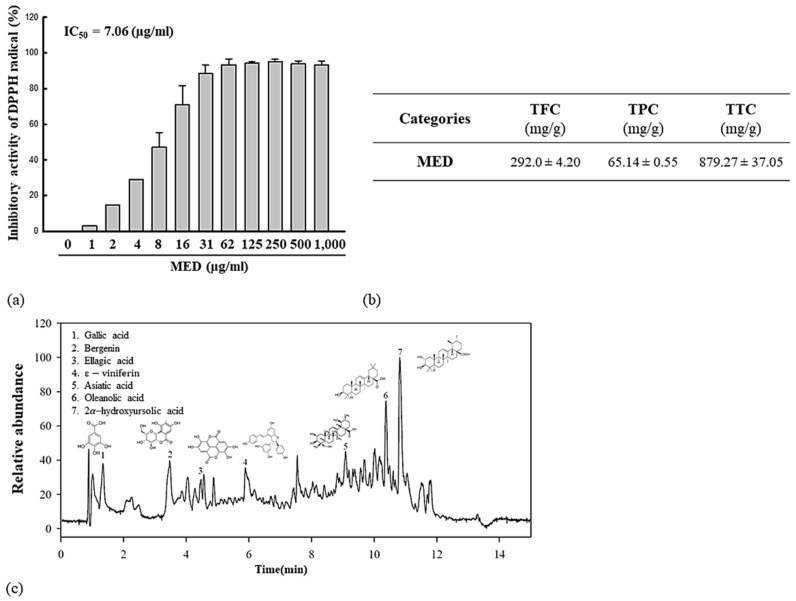
Phytochemical composition and free radical scavenging activity of MED. (**a**) DPPH radical scavenging activity was measured in a mixture including 0.1 mM DPPH and varying concentrations of MED (1–1000 μg/mL). Three MED samples were assayed in duplicate by DPPH radical scavenging activity analysis. Data are reported as the mean ± SD. (**b**) TFC, TPC, and TTC were determined at different concentrations of MED. Data are reported as the mean ± SD. (**c**) LC-MS analysis of MED. Seven active components including gallic acid, bergenin, ellagic acid, ε-viniferin, asiatic acid, oleanolic acid, and 2α-hydroxyursolic acid were detected as each different peak in chromatogram. Abbreviations: LC–MS/MS, liquid chromatography tandem mass spectrometry; IC_50_, half maximum inhibitory concentration; TFC, total flavonoid content; TPC, total phenol content; TTC, total condensed tannin.

To measure the antioxidant activity of MED, we measured the DPPH scavenging activity at various concentrations of MED. Dose-dependent inhibitory activity against DPPH radicals was observed at 1–62 µg/mL MED, and the IC_50_ value was determined to be 7.06 µg/mL (Figure 1a). Moreover, the phytochemical composition of was analyzed in MED. High levels of total condensed tannin and total phenol were detected (879.27 and 292.0 mg/g, respectively), whereas the total flavonoid contents were determined to be 65.14 mg/g (Figure 1b). Furthermore, LC-MS analyses were performed to identify and characterize active components. In particular, gallic acid, bergenin, ellagic acid, ε-viniferin, asiatic acid, oleanolic acid, and 2α-hydroxyursolic acid were detected in MED (Figure 1c). These results indicate that MED exhibits strong anti-oxidative activity and has the potential for application as an anti-photoaging compound with high antioxidant activity.

### 3.2. Recovery Effect of MED on UV-Induced NHDF Cell Death

We next investigated the inhibitory effects of MED on UV-induced NHDF cell death. To achieve this, we first determined the optimal conditions of UV and MED in NHDF cells. Cell viability was remarkably decreased subsequent to UV radiation between 45 and 65 mJ/cm^2^, with 70% decrease observed in cells exposed to 55–65 mJ/cm^2^ (Supplementary Figure S1). However, treatment with MED at 100, 200, and 400 µg/mL for 24 h caused no significant cell death (Supplementary Figure S2). On the basis of the above results, we decided the optimal dosages of UV radiation and MED at 55 mJ/cm^2^ and 100, 200, and 400 µg/mL, respectively.

**Figure 2:**
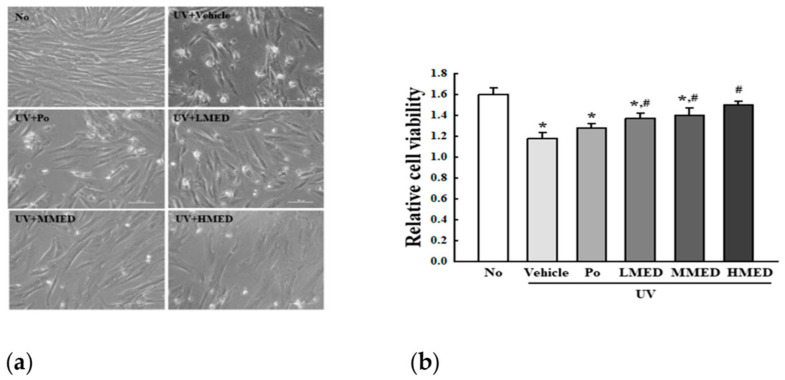
Cytotoxicity of UV + MED-treated NHDF cells. (**a**) After incubation of UV-radiated NHDF cells with 100, 200, and 400 μg/mL MED for 24 h, cell morphological changes were observed under a microscope at 400× magnification. (**b**) Two to three wells per group were used for the MTT assay, and optical density was measured in duplicate. Data are reported as the mean ± SD. * *p* < 0.05 relative to the No-treated group. # *p* < 0.05 compared to the UV + Vehicle-treated group.

To investigate the recovery effect of MED on UV-induced cell death, we measured cell viability after treating cells with 55 mJ/cm^2^ UV and 100, 200, and 400 µg/mL MED. Compared to the no group, viabilities of NHDF cells were lower in the UV-treated group, with about 30% viability in the UV + Vehicle-treated group. However, the viabilities were dose-dependently increased in the UV + LMED, UV + MMED, and UV + HMED-treated groups (Figure 2). These results indicate that MED treatment helps in the recovery of UV-induced NHDF cell death.

### 3.3. Enhancement Effect of MED on Antioxidative Activity

**Figure 3:**
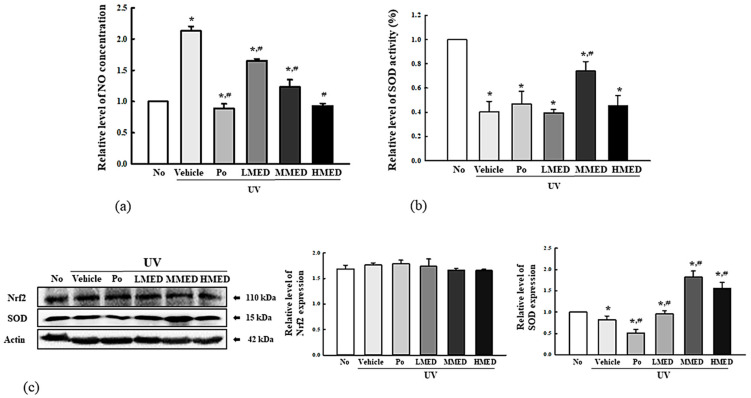
Antioxidant activity of MED-treated NHDF cells. (**a**) Determination of NO concentration. NHDF cells were treated with the vehicle, LMED, MMED, and HMED in the absence or presence of UV radiation for 24 h. NO concentration was measured in the culture supernatants using Griess reagent. Data are reported as the mean ± SD. (**b**) Determination of SOD activity. SOD activity in lysate of NHDF cells was detected in each subset group. One SOD unit is represented as the amount of the enzyme in the MED solution (20 µL) that inhibits the reduction reaction of water-soluble tetrazolium salt-1 (WST-1) with superoxide anion by 50%. (**c**) Detection of SOD and Nrf2 expression. Total cell lysates were prepared from NHDF cells after treatment of UV + MED. Expression levels of the two proteins were detected with specific antibodies and quantified using an imaging densitometer. Two to three samples were analyzed in duplicate by Western blotting. Data are reported as the mean ± SD. * *p* < 0.05 compared to the No-treated group. # *p* < 0.05 compared to the UV + Vehicle-treated group. Abbreviations: SOD, superoxide dismutase; Nrf2, nuclear factor erythroid 2-related factor 2.

To investigate whether the recovery effect of MED on UV-induced cell death was associated with its antioxidative activity in NHDF cells, we measured for alterations in the NO concentration, SOD activity and expression, and Nrf2 expression in UV + MED-treated cells. NO concentration was 2.2 times higher in the UV + Vehicle-treated NHDF cells, although there was no morphologic change. However, these levels were significantly and dose-dependently decreased in the UVB + MED-treated groups (Figure 3a). SOD activity and expression were decreased in the UV + Vehicle-treated group, but levels were significantly enhanced in the UV + MED-treated groups, although its activity was higher in only the UV + MMED group (Figure 3b,c). However, the expression level of Nrf2 proteins was constantly maintained in in the UV + Vehicle group and UV + MED-treated groups (Figure 3c). Taken together, these results indicate that the high SOD activity of MED is tightly associated with its recovery effects on UV-induced cell death.

### 3.4. Inhibitory Effects of MED on Apoptosis of UV-Irradiated NHDF Cells

**Figure 4:**
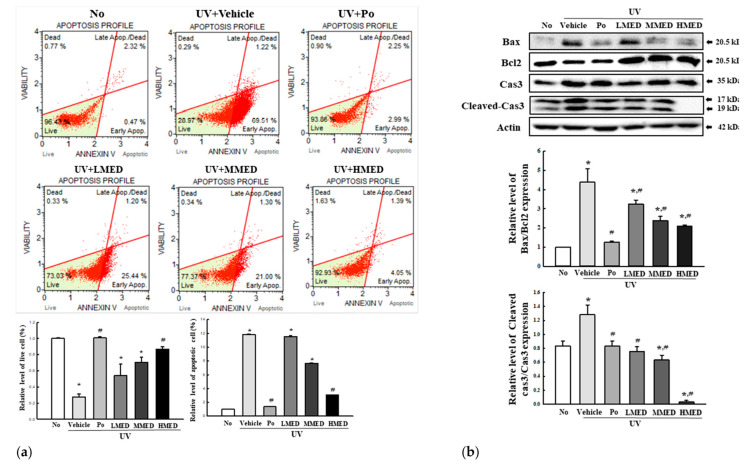
Apoptosis analysis of UV + MED-treated NHDF cells. (**a**) Analysis of annexin V-stained NHDF cells. After treatment with LMED, MMED, and HMED for 24 h, the distribution of cells was analyzed subsequent to staining with annexin V and 7-AAD. Initial cell population gating was placed on cell size vs. annexin V. Subsequently, the most obvious debris was gated out from the total cell population. Two to three wells per group were used for annexin V staining, and the number of dead cells and live cells was measured in duplicate. (**b**) Expression of apoptotic proteins. After preparation of total cell lysates, the expression levels of apoptotic proteins, including Bax, Bcl-2, Cas3, cleaved Cas3, and β-actin, were measured by Western blot analysis. Three to four wells per group were used in the preparation of the total cell lysate, and Western blot analyses were assayed in duplicate for each sample. Data are reported as the mean ± SD. * *p* < 0.05 compared to the No-treated group. # *p* < 0.05 compared to the UV + Vehicle-treated group. Abbreviations: 7-AAD, 7-aminoactinomycin D.

To determine whether the recovery effects of MED on UV-induced cell death is related to the regulation of apoptosis, we counted apoptotic and live cells after staining with the annexin V/PI detection kit, and expression levels of apoptotic proteins were measured with Western blot analysis. The total number of apoptotic cells was remarkably increased in the UV + Vehicle-treated group, as compared to the no group; moreover, total live cells were decreased by about 50% in the same group. However, the number of apoptotic cells and live cells were significantly decreased or increased, respectively, in a dose-dependent manner in the UV + MED-treated groups (Figure 4a). Furthermore, alterations in the apoptotic cell numbers were entirely reflected in the expression of proteins related to apoptosis regulation. Increased levels of Bax/Bcl2 expression were dramatically decreased in a dose-dependent manner after treatment with MED. Similar expression patterns were observed for levels of cleaved Cas-3/Cas-3 expressions in the UV + LMED-, UV + MMED-, and UV + HMED-treated groups (Figure 4b). These results indicate that the recovery effects of MED are associated with the inhibition of apoptosis in UV-induced cells. In addition, the recovery effects of MED on UV-induced cell death are probably associated with increased G0/G1 arrest (Supplementary Figure S3).

### 3.5. Recovery Effect of MED on Galactosidase, Elastase, and Collagenase Level in UV-Radiated NHDF Cells

**Figure 5:**
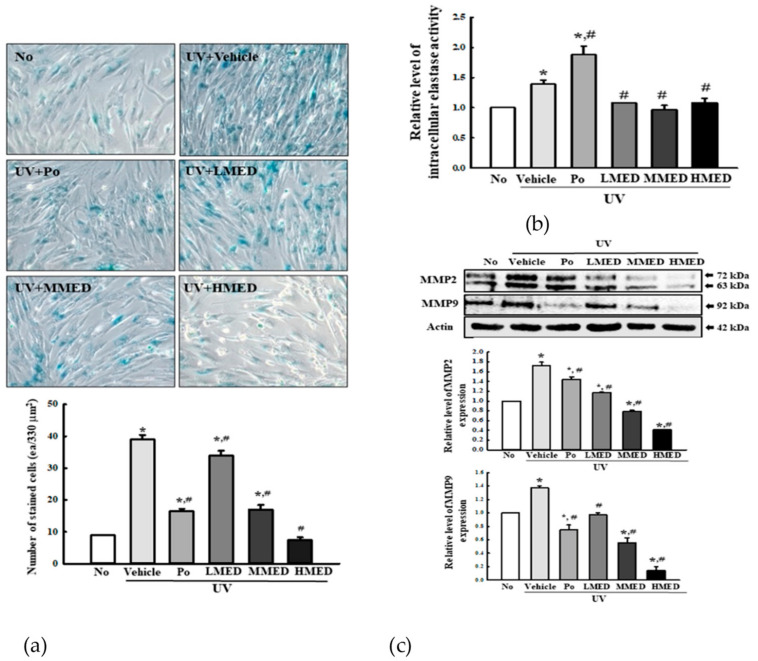
Galactosidase staining, intracellular elastase analyses, and collagenase expression. (**a**) Galactosidase staining analyses. NHDF cells were incubated with galactosidase staining solution at 37 °C; the stained cells were microscopically observed at 400× magnification. Blue color indicates the X-gal-stained cell. Two to three dishes per group were prepared for X-gal staining analysis, and cell number was assayed in duplicate. (**b**) Intracellular elastase activity analyses. After collection of cell lysate, enzyme activity was determined by applying the inhibition rate of elastase, as described in the Materials and Methods section. Two to three dishes per group were prepared for cell lysate, and samples were assayed in duplicate. (**c**) MMP2 and -9 expressions. The level of MMP2, MMP9, and β-actin proteins in lysates were determined by Western blotting. The intensity of each band was measured using an imaging densitometer. The relative levels of MMP-2/9 proteins were calculated in comparison with β-actin protein. Two to three dishes per group were combined for Western blot analysis, and samples were assayed in duplicate. Data are reported as the mean ± SD. * *p* < 0.05 compared to the No-treated group. # *p* < 0.05 compared to the UV + Vehicle-treated group.

β-Galactosidase is regarded as one of the biomarkers for cellular senescence because this enzyme is capable of hydrolyzing β-galactoside into monosaccharides only in senescent cells. To investigate the recovery effects of MED on biomarkers of skin aging, we measured the β-galactosidase level in UV + MED-treated NHDF cells. Increased β-galactosidase staining after UV radiation was remarkably recovered in a dose-dependent manner in the UV + MED-treated groups (Figure 5a). Moreover, this recovery of β-galactosidase was completely reflected in the levels of elastase and collagenase. The intracellular elastase activity and expressions of MMP2/9 were remarkably decreased in the three UV + MED-treated groups, as compared to the UV + Vehicle-treated group (Figure 5b,c). These results indicate that MED inhibits the skin aging of UV-treated NHDF cells by suppressing the levels of β-galactosidase, elastase, and collagenase.

### 3.6. Suppression Effect of MED on the Inflammatory Response in UV-Radiated NHDF Cells

**Figure 6:**
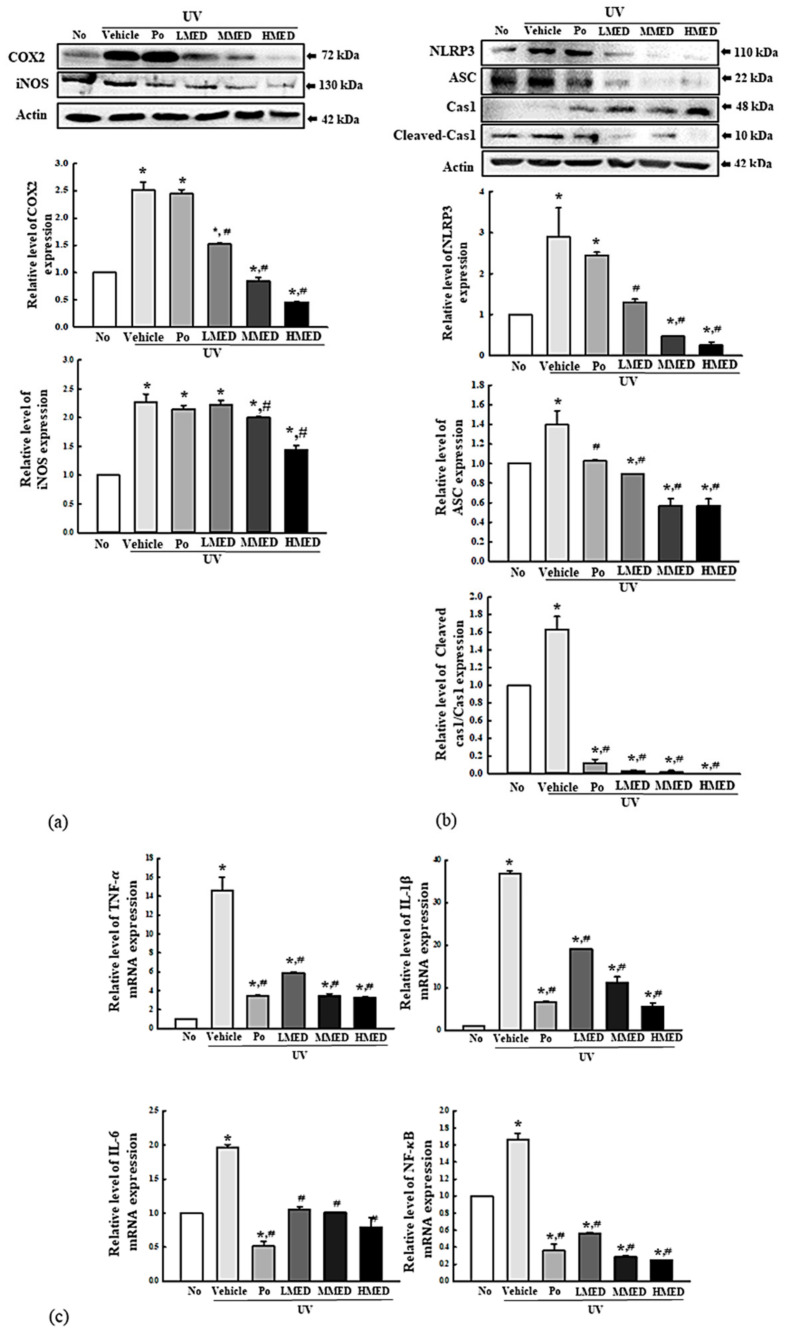
Expression of inflammatory mediators. (**a**) iNOS-induced COX2-mediated pathway analysis. After preparation of total cell lysates and RNA, the expression levels of inflammatory proteins and cytokines were measured by Western blot analysis, using specific antibodies and real-time PCR analysis. (**b**) Expression of inflammasome proteins. Using specific antibodies, Western blot was performed to detect ASC, Cas1, and NLRP3 proteins in the homogenates of UV-radiated NHDF cells treated with MED. Three to four wells per group were used in the preparation of total homogenate, and Western blot analyses were assayed in duplicate for each sample. (**c**) Expression of inflammatory cytokines. After preparation of total RNA from skin tissue, the mRNA levels of TNF-α, IL-6, IL-1β, and NF-κB were measured by RT-qPCR using specific primers. Data are reported as the mean ± SD. * *p* < 0.05 compared to the No-treated group. # *p* < 0.05 compared to the UV + Vehicle-treated group.

The inflammatory response, including iNOS-induced COX2-mediated pathway and NLR family pyrin domain containing 3 (NLRP3) inflammasome, plays a crucial role in keratinocytes during UV-induced photoaging [22,23,24]. We investigated the suppressive effects of MED on the inflammatory response in UV-radiated NHDF cells. To achieve this, alterations in the iNOS-induced COX2-mediated pathway and inflammasome were analyzed in the UV + MED-treated NHDF cells. The expression levels of iNOS and COX2 were significantly decreased in the UV + MED-treated group, whereas increased levels of these proteins were detected in the UV + Vehicle-treated group (Figure 6a). Moreover, the regulation of iNOS and COX2 expression was completely reflected in inflammasome activation. The expression levels of NLRP3 and apoptosis-associated speck-like protein containing CARD (ASC) proteins were higher in the UV + Vehicle-treated group than in the untreated group. However, these levels were remarkably recovered in a dose-dependent manner after treatment of MED, although the ASC level was constantly maintained in the UV + MMED- and UV + HMED-treated groups. A similar pattern was observed for cleaved Cas1/Cas1 expression. These values were significantly decreased in the UV + MED-treated groups (Figure 6b). Expressions of inflammatory cytokines were also observed to follow a similar pattern. The UV + MED-treated groups showed significant decrease in the TNF-α, IL-6, IL-1β, and NF-κB mRNA levels, as compared to the UV + Vehicle-treated group (Figure 6c). These results indicate that MED contributes to the suppression of UV-induced inflammatory response in NHDF cells through regulation of the iNOS-induced COX2-mediated pathway and NLRP3 inflammasome activation.

### 3.7. Recovery Effects of MED on Phenotypes and Histological Structure of Skin in Nude Mice

**Figure 7:**
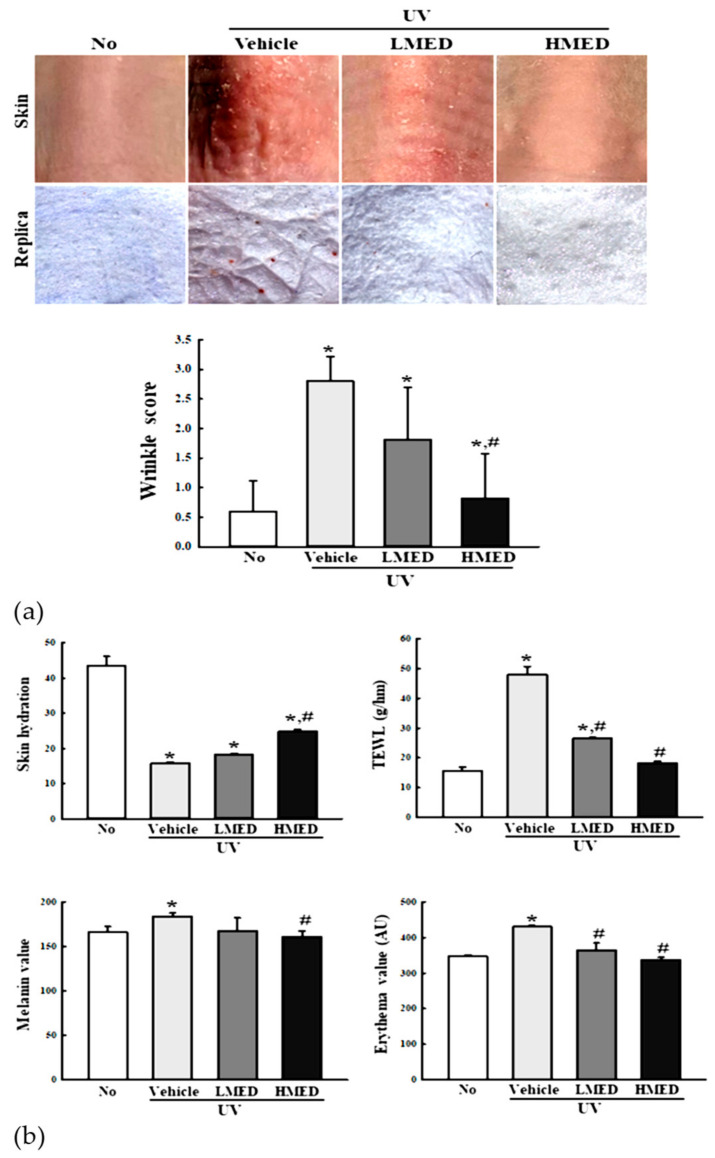
Skin phenotypical analyses of UV + MED-treated mice. (**a**) Wrinkle scores were determined on the skin using the method suggested by Bissett et al. (grade 0, no wrinkles; grade 1, a few shallow wrinkles; grade 2, some wrinkles; grade 3, several deep wrinkles). (**b**) TEWL, skin hydration, and erythema and melanin indices were determined in dorsal skin in triplicate. Three to five mice per group were used, and two different dorsal skin areas per mouse were assayed in duplicate. Data are reported as the mean ± SD. * *p* < 0.05 compared to the No-treated group. # *p* < 0.05 compared to the UV + Vehicle-treated group. Abbreviations: TEWL, trans-epidermal water loss.

**Figure 8:**
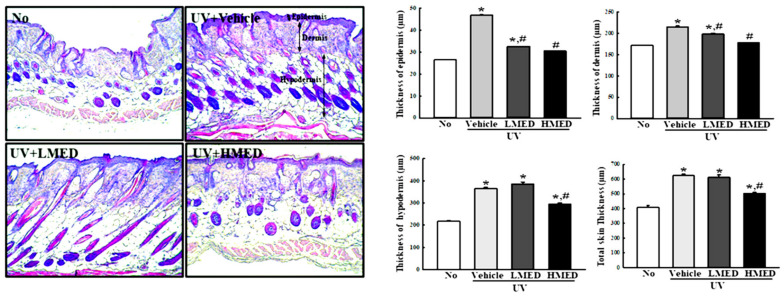
Histopathological structures in the skin of UV + MED-treated mice. The dorsal skin tissues of nude mice were fixed in 4% formaldehyde solution. Alterations in the histopathological structures in skins obtained from UV + MED-treated mice were observed microscopically at 200× magnification after staining with H&E solution. Thickness of the epidermis, dermis, hypodermis, and total skin were assayed on H&E-stained slides in duplicate for each mouse. Three to five mice per group were used for the preparation of H&E-stained tissue, and thickness of skin tissue was measured in duplicate. Data are reported as the mean ± SD. * *p* < 0.05 compared to the No-treated group. # *p* < 0.05 compared to the UV + Vehicle-treated group.

To assess applicability to humans, we consequently verified the effectiveness of MED in NHDF cells in an animal model. We first evaluated the recovery effects of MED on the phenotypes and histological structure of skin in UV-irradiated nude mice. The nude mice were decided on the basis of results from previous research for anti-photoaging activity of biomaterials and natural products because this mouse can evaluate both skin protection and anti-inflammation effects of these substances at the same time [25,26,27,28]. The wrinkle scores, which included consideration of wrinkle depths and numbers, were remarkably enhanced in the UV + Vehicle-treated group, but these enhancements were significantly decreased in the UV + MED-treated groups (Figure 7a). Moreover, MED administration stimulated a significant recovery of UV-induced changes on TEWL, skin hydration, erythema value, and melanin value. The decreased level of skin hydration in the UV + Vehicle-treated group was significantly increased in the UV + MED-treated groups (Figure 7b). Similar recovery effects were observed in histological structures, including thickness of epidermis, dermis, hypodermis, and total skin (Figure 8). These results indicate that MED administration contributes to the recovery of UV-induced alterations on phenotypes and the histological structure of mice skin.

### 3.8. Antioxidative Activity of MED in UV-Radiated Skin

**Figure 9:**
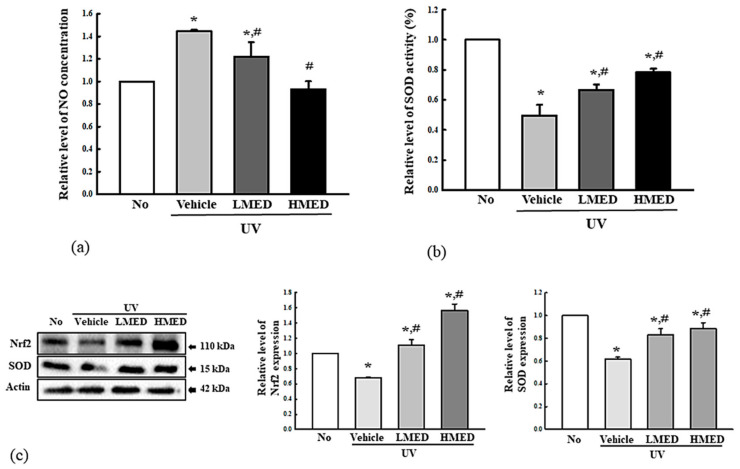
Antioxidant activity of MED-treated nude mice. (**a**) Determination of NO concentration. Nude mice were treated with Vehicle, LMED or HMED in the presence of UV radiation for 4 weeks. After collecting the skin tissue, we measured NO concentration using Griess reagent. (**b**) Determination of SOD activity. SOD activity in homogenates of skin tissue was detected in each subset group. One SOD unit is represented as the amount of the enzyme in the MED solution (20 µL) that inhibits the reduction reaction of water-soluble tetrazolium salt-1 (WST-1) with superoxide anion by 50%. (**c**) Detection of SOD and Nrf2 expression. Total tissue lysates were prepared from nude mice after treatment of UV + MED. Expression levels of the two proteins were detected with specific antibodies and quantified using an imaging densitometer. Three to five mice per group were used for the preparation of tissue lysate, and NO assay, SOD activity assay, and Western blotting were measured in duplicate. Data are reported as the mean ± SD. * *p* < 0.05 compared to the No-treated. # *p* < 0.05 compared to the UV + Vehicle-treated group. Abbreviations: SOD, superoxide dismutase; Nrf2, nuclear factor erythroid 2-related factor 2.

To investigate whether the recovery effect of MED on UV-induced skin photoaging is associated with its antioxidative activity, we measured changes in the NO concentration, SOD activity and expression, and Nrf2 phosphorylation in the skin of UV + MED-treated mice. NO concentration was higher at 45% in the UV + Vehicle-treated group. However, these levels were dose-dependently decreased in the UV + MED-treated groups (Figure 9a). Conversely, the decreased SOD activity and expression in the UV + Vehicle-treated group was increased after exposure in the UV + MED-treated groups (Figure 9b,c). Furthermore, the recovery effect on Nrf2 expression was observed in the UV + MED-treated groups, as compared to the UV + Vehicle-treated groups (Figure 9c). Taken together, these results suggest that the antioxidant activity of MED is probably associated with its recovery effects on UV-induced skin photoaging.

### 3.9. Inhibitory Effects of MED on UV-Induced Apoptosis in Mice Skin

**Figure 10:**
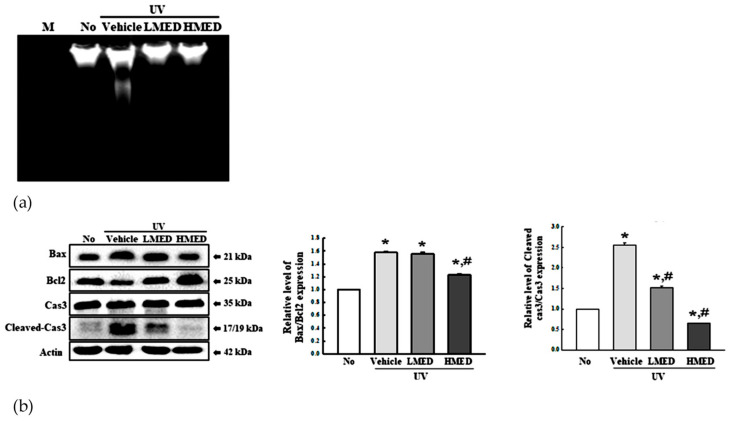
Apoptosis analysis in UV + MED-treated nude mice. (**a**) DNA fragmentation assay. Fragmented DNA was detected on agarose gel electrophoresis of DNA isolated from the skin tissue of subset groups. Three to four skins per group were used in the preparation of the genomic DNA, and electrophoresis was performed in duplicate for each sample. (**b**) Expression of apoptotic proteins. After preparation of total tissue lysates, the expression levels of apoptotic proteins, including Bax, Bcl-2, Cas3, cleaved Cas3, and β-actin, were measured by Western blot analysis, using specific antibodies. Three to four mice per group were used in the preparation of the total homogenate, and Western blot analyses were assayed in duplicate for each sample. Data are reported as the mean ± SD. * *p* < 0.05 compared with the No-treated group. # *p* < 0.05 compared to the UV + Vehicle-treated group. Abbreviations: M, marker.

To determine whether the recovery effects of MED on UV-induced skin photoaging are accompanied with inhibition of apoptosis, we measured the level of fragmented DNA and apoptotic proteins in the skin of UV + MED-treated mice. A significant increase of fragmented DNA was observed in the UV + Vehicle-treated group, as compared to the no group, with remarkable recovery of these levels in the UV + MED-treated group (Figure 10a). Similar effects of MED were detected in the ratio of Bax/Bcl2 and cleaved Cas3/Cas3 expressions in the UV + MED-treated groups (Figure 10b). These results indicate that the recovery effects of MED are probably associated with the inhibition of UV-induced apoptosis in mice skin.

### 3.10. Recovery Effect of MED on Elastase and Collagenase Levels of UV-Radiated Skin

**Figure 11:**
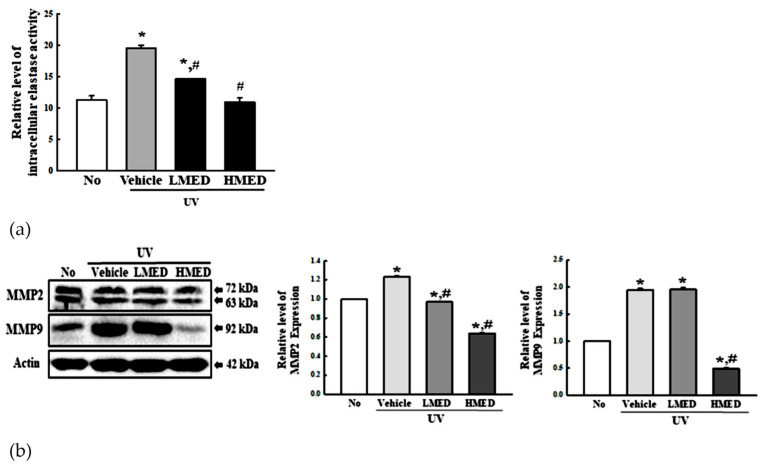
ECM modulation analysis. (**a**) Intracellular elastase activity analyses. After collection of cell lysate, enzyme activity was determined using inhibition rate of elastase, as described in the Materials and Methods section. Two to three mice per group were used for preparing the total homogenates, and enzyme activity was assayed in duplicate. (**b**) Expression of MMP. After preparation of total tissue homogenate, the expression levels of MMP2, MMP9, and β-actin were measured by Western blot analysis using specific antibodies. Three to four mice per group were used in the preparation of the total homogenate, and Western blot analyses were assayed in duplicate for each sample. Data are reported as the mean ± SD. * *p* < 0.05 compared with the No-treated group. # *p* < 0.05 compared to the UV + Vehicle-treated group.

To determine whether the recovery effects of MED on UV-induced skin photoaging are accompanied with alterations in the levels of elastase and collagenase, we measured for changes in the intracellular elastase activity and MMP2/9 expression in the UV + MED-treated mice. Increase of intracellular elastase activity after UV radiation was remarkably recovered in a dose-dependent manner in the UV + MED-treated groups (Figure 11a). A similar recovery pattern was detected for the expressions of MMP2 and -9. These levels were completely recovered to normal in all three UV + MED-treated groups, although a dramatic change was observed in the expression level of MMP9 (Figure 11b). These results indicate that the recovery effects of MED during UV-induced skin photoaging are associated with the suppression of intracellular elastase activity and MMP expressions.

### 3.11. Suppression Effect of MED on the Inflammatory Response in UV-Radiated Skin

**Figure 12:**
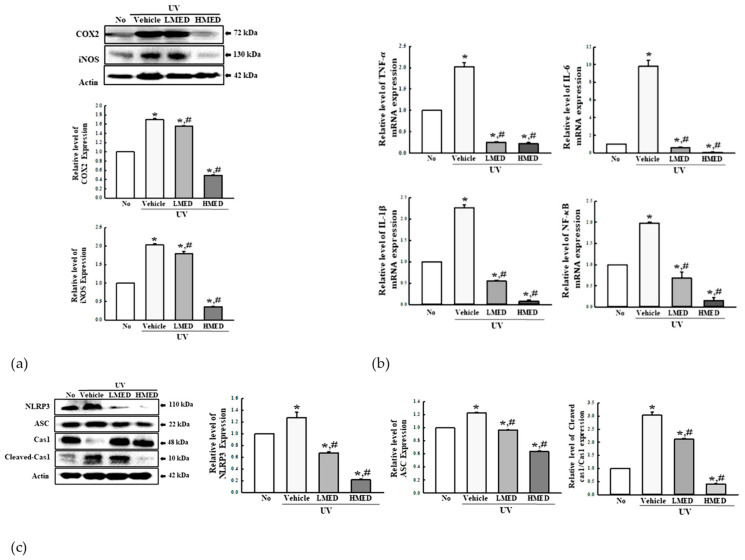
Analyses of inflammatory markers. (**a**) Expressions of COX2 and iNOS proteins. After preparation of total tissue lysates, the expression levels of COX2, iNOS, and β-actin were measured by Western blot analysis, using specific antibodies. (**b**) mRNA levels of inflammatory cytokines. After preparation of total RNA from skin tissue, the mRNA levels of TNF-α, IL-6, IL-1β, and NF-κB were measured by RT-qPCR, using specific primers. (**c**) Expression of inflammasome proteins. After preparation of total tissue lysates, the expression levels of NLRP3, ASC, Cas1, cleaved Cas1, and β-actin were measured by Western blot analysis, using specific antibodies. Three to four mice per group were used in the preparation of total RNA and tissue homogenates, Western blot and RT-qPCR were analyzed in duplicate for each sample. Data are reported as the mean ± SD. * *p* < 0.05 compared with the No-treated group. # *p* < 0.05 compared to the UV + Vehicle-treated group.

Finally, we investigated whether the recovery effects of MED on UV-induced skin photoaging is accompanied with suppression of the inflammatory response. To achieve this, alterations in the iNOS-induced COX2-mediated pathway, inflammatory cytokines, inflammasome activation, and mast cells infiltrations were measured in the skin tissue of UV + MED-treated nude mice. The increased levels of COX2 and iNOS proteins in the UV + Vehicle-treated group were significantly decreased in the UV + MED-treated mice, with dramatic decrease detected only in the UV + HMED-treated group (Figure 12a). A similar decrease pattern was observed for inflammatory cytokine expressions, including TNF-α, IL-6, IL-1β, and NF-κB (Figure 12b). Furthermore, significant suppression of the inflammasome activation was observed in the skin of UV-radiated mice after MED administration. The expression levels of NLRP3, ASC, and cleaved Cas3 proteins were remarkably decreased in the UV + LMED- and UV + HMED-treated groups (Figure 12c). The infiltration of mast cells into the dermis region was suppressed in the skin of UV-radiated mice after MED administration (Supplementary Figure S4). Taken together, these results indicate that the recovery effects of MED during UV-induced skin photoaging is associated with suppression of the inflammatory response, including the iNOS-induced COX2-mediated pathway, inflammatory cytokines, inflammasome activation, and mast cell infiltrations.

## 4. Discussion

Antioxidants are considered an important strategy in the treatment of skin photoaging since UV radiation induces serious oxidative stress, including the overproduction of ROS and decrease of endogenous antioxidants [29,30]. Considering this, some natural products with high antioxidant activity are well known as key sources for investigating the therapeutic effects and mechanism of action in several cell lines and in skin of animal models [8,10,11,12]. As part of a study aimed at identifying novel natural products with antioxidative effects, we focused on the therapeutic effects and mechanism of MED in skin fibroblasts and skin tissue of nude mice after UV irradiation. Results of the present study provide novel scientific evidence that MED administration contributes to the suppression of UV-induced skin photoaging through regulation of the antioxidant activity, apoptosis, aging makers, and inflammatory response. Since the current study solely applied the methanol extract of MED, further studies are required to verify the correlation between key components of MED and the protective mechanism imparted on UV-induced photoaging. In this study, we identified seven active components, namely, gallic acid, bergenin, ellagic acid, ε-viniferin, asiatic acid, oleanolic acid, and 2α-hydroxyursolic acid, in MED during LC-MS analysis. These components are known to be distributed on the stem and baker of in genus *Dipterocarpus*, although their species were varied [31]. Bergenin was found in stem and bark of *D. tuberculatus* and it showed an antioxidative activity by quenching free radicals, of which methoxyl group (O-6–CH_3_) is the most favorable site for radical attack [31,32]. ε-Viniferin, a resveratrol a dehydrodimer with a five-membered oxygen heterocyclic ring, is a naturally occurring phenol, belonging to the stilbenoids family. It has been reported that ε-viniferin has more effective antioxidant and anti-inflammatory activities than resveratrol [33]. Oleanolic acid and 2α-hydroxyursolic acid are triterpenes presented in several herbs. Among these, oeanolic acid has been reported to exhibit a dose-dependent effect in superoxide anion scavenging activity, chelating effect, and xanthine oxidase inhibition activity [34]. 2α-Hydroxyursolic acid is known to have potent antioxidative activities in DPPH free radical scavenging and superoxide anion scavenging [35]. Therefore, our results on the LC-MS chromatogram suggest that four components identified in MED are highly likely to be the main active components for anti-photoaging effects.

To date, few studies have analyzed the correlation between natural products and antioxidative activity to elucidate their therapeutic effects against UV-induced photoaging. Grapefruit extracts (12.5–100 μg/mL) inhibit the UV-induced harmful effects on human HaCaT keratinocytes and in human volunteers through decrease of UVB-induced intracellular ROS production [11]. Moreover, garlic possesses strong DPPH radical (IC_50_ = 2.50 mg/mL) and NO scavenging activity (4.38 mg/mL), and is reported to inhibit UVB-induced photoaging in HaCaT human keratinocytes [36]. A similar anti-photoaging activity was observed in NHDF cells and the hairless mouse model treated with *Foeniculum vulgare*. After exposure to this extract, UVB-induced intracellular ROS production was inhibited, with simultaneous increase in the levels of antioxidant glutathione (GSH) [37]. Furthermore, the green tea seed extract showed suppressive effects on UVB-induced MMP expressions and activity of antioxidant enzymes [38]. In the current study, we analyzed the levels of NO concentration, SOD activity, and MMP expression in NHDF cells and nude mice in order to determine whether the antioxidative ability of MED is the main cause of anti-photoaging effects. Our results are similar to those obtained in previous studies, although there are differences in analysis factors and their effectiveness. Therefore, the results of this MED study provide additional evidence that natural products with high antioxidant activity are potentially important therapeutic drugs against UV-induced photoaging. Especially, the alteration in the expression level of Nrf2 was perfectly consistent with the alteration in SOD activity and expression level in UV + MED-treated nude mice, and they did not match in NHDF cells. Actually, Nrf2 is well known as one of key regulators for redox homeostasis because it dissociated from the Keap1–Nrf2 complex can promote the transcription of many antioxidant and detoxification genes through binding to the antioxidant response element (ARE) during oxidative stress [39]. In normal physiological conditions, this complex in the cytosol is proteasomal degraded by the ubiquitination to maintain the redox homeostasis [39,40]. Moreover, the downregulation of Nrf2 expression level in UV-treated hairless mice was significantly recovered with treatment of some products including fisetin and skin-derived precursor cells [41,42]. These results are in agreement with the results of present study, where the Nrf2 expression is shown to be decreased after treatment to MED for 4 weeks in nude mice. In addition, our results in NHDF cells are consistent with previous studies. UVA and UVB irradiation did not induce any significant alteration in Nrf2 expression in keratinocytes, although the levels of intracellular ROS enhanced [43]. Therefore, further research is needed to advance our understanding of causes for the differences of Nrf2 expression between cells and mice.

We examined the anti-photoaging effects of MED on UV-radiated skin of nude mice. Oral administration of MED inhibited the UV-induced alterations on wrinkle formation, TEWL, skin hydration, and erythema and melanin indices. The recoveries on these markers for skin photoaging have similarly been observed in the UV-radiated mice model after oral administration of several natural products, although there are differences in methods of analysis and their effective outcomes. Extract of the fermented *Cyclopia intermedia* (honeybush) improved skin wrinkles, elasticity, and hydration in 120 Korean subjects with crow’s feet wrinkles, while cold-pressed perilla oil inhibited an increase in wrinkle formation, TEWL, erythema value, hydration, and melanin index on the dorsal skin of UVB-irradiated hairless mice [44]. Moreover, a similar improvement was observed for wrinkle formation, epidermal thickening, erythema, hyperpigmentation, skin hydration, and TEWL in UVB-irradiated hairless mice after oral administration of *Agastache rugosa* [45]. Furthermore, several extracts, including the dietary enzyme-treated *Hibiscus syriacus* and Hochuekkito, ameliorated wrinkle formation, TEWL, stratum corneum (SC) hydration, and erythema index in hairless mice after UVB exposure [46,47]. Meanwhile, oral route and topical route are widely used as animal treatment methods to assess anti-photoaging effects of natural products. The former has the advantage such as high bioavailability, fast absorption, and high anti-inflammatory effect, although it has first pass effects [48]. Topical route of administration exhibits good hydration effect and avoiding first pass effect, and is being considered as a method to allow long-term exposure without any significant side effects, while it has some disadvantages including low permeability, different penetration efficacy, and taking a long time to approach the blood [48]. On the basis of above properties, researchers alternatively selected these two routes as the optimal method in various studies in order to examine anti-photoaging effects of natural products [48]. Actually, the efficacy of one natural product has not been directly compared in two routes. However, the similar anti-photoaging effect of the same natural product such as *P. leucotomos*, green tea, and ginseng was detected in animals treated with two different routes [49,50,51,52,53].

Along with the antioxidant activity, MED administration also imparted anti-inflammatory effects on UV-radiated NHDF cells and skin of nude mice, which was validated by determining the suppression of mast cell infiltrations, iNOS-induced COX2-mediated pathway, and inflammasome activation. In particular, HMED-treated mice showed complete inhibition of iNOS, COX2, inflammatory cytokines, NLRP3, ASC, and cleaved Cas1 expression levels in UV-radiated NHDF cells and mice skin. Among the above analyzing mechanisms, the iNOS-induced COX2-mediated pathway is considered a key regulatory mechanism wherein the balance of oxidative inhibitors and stimulators in the body is disturbed by events such as inflammation. In various inflammatory diseases, the iNOS and COX2 proteins in this pathway are induced by a variety of pro-inflammatory stimuli such as TNF-α and LPS. Moreover, the overexpression and activation of iNOS promote the production of NO, which stimulates the activation of COX2. Furthermore, the iNOS-induced COX2-mediated pathway is mediated by the MAPK signaling pathway, which plays a critical role in the regulation of differentiation and cell growth as well as in the regulation of cellular responses against stresses and cytokines. Some natural products, including *Ixora parviflora*, *Aspalathus linearis*, *Citrus sinensis*, *Cyclopia* spp., *Foeniculum vulgare*, and *Glycine max*, inhibit the NO production and COX2 expression as well as inflammatory cytokine expressions during UV-induced photoaging [37,54,55,56].

Inflammasome is a multiprotein cytosolic complex and intracellular sensor that detects a broad range of pathogenic microorganisms during the innate immunity response [57,58]. Pattern recognition receptors (PRRs) and damage-associated molecular patterns (DAMPs) of microorganisms have been recognized with this complex [59]. Moreover, a nucleotide-binding oligomerization domain-like receptor (NLR) protein is the main component of inflammasomes and is classified into four types: NLRP1/NALP1b inflammasome [60], NLRC4/IPAF inflammasome [61,62], NLRP3/NALP3 inflammasome [63], and AIM2 (absent in melanoma 2) containing inflammasome [64]. However, inflammasomes were not considered as potential targets in molecular mechanisms and therapeutic strategies for UV-induced photoaging until now. Only one study has previously investigated the role of natural products in UV-induced inflammasome activation. The combination of fucoxanthin (FX) and rosmarinic acid (RA) resulted in decreased expressions in the levels of inflammasome components such as NLRP3, ASC, and Cas1 in UVB-irradiated HaCaT keratinocytes [65]. Similarly, our results also suggest the possibility that inflammasome is a potentially important target for evaluating the efficacy of natural products with high antioxidative activity on UV-induced photoaging.

## 5. Conclusions

The current study measures the antioxidant activity of MED as well as alterations in anti-apoptosis, ECM modulation, skin appearance, and anti-inflammatory response in UV-radiated NHDF cells and skin tissue of nude mice treated with MED. The results of the present study demonstrate that the high antioxidant activity of MED successfully suppresses the apoptotic proteins, inflammasome activation, MMP expressions, wrinkle score, skin phenotypes, and histopathological structure. Therefore, the recovery effects of MED on UV-induced damages of NHDF cells and nude mice indicates the potential of MED as an anti-photoaging drug.

## Data Availability

All the data that support the findings of this study are available on request from the corresponding author.

## Supplementary Materials

The following are available online at https://www.mdpi.com/article/10.3390/antiox10050791/s1, Figure S1: Determination of optimal UV dosage, Figure S2: Cytotoxicity of MED, Figure S3: Cell cycle analysis of MED treated NHDF cells, Figure S4: Infiltration of mast cells, Table S1: Antibodies list for Western blot analyses, Table S2: Primer sequence for RT-PCR.

## References

[1] Fisher G.J., Kang S., Varani J., Bata-Csorgo Z., Wan Y., Datta S., Voorhees J.J. (2002). Mechanisms of photoaging and chronological skin aging. Arch. Dermatol..

[2] Dickinson B.C., Chang C.J. (2011). Chemistry and biology of reactive oxygen species in signaling or stress responses. Nat. Chem. Biol..

[3] Corsini E., Sangha N., Feldman S.R. (1997). Epidermal stratification reduces the effects of UV-B (but not UV-A) on keratinocyte cytokine production and cytotoxicity. Photodermatol. Photoimmunol. Photomed..

[4] Pillai S., Oresajo C., Hayward J. (2005). Ultraviolet radiation and skin aging: Roles of reactive oxygen species, inflammation and protease activation, and strategies for prevention of inflammation-induced matrix degradation. Int. J. Cosmet. Sci..

[5] Rabe J.H., Mamelak A.J., McElgunn P.J.S., Morison W.L., Sauder D.N. (2006). Photoaging: Mechanisms and repair. J. Am. Acad. Dermatol..

[6] Chen D., Du Z., Lin Z., Su P., Huang H., Ou Z., Pan W., Huang S., Zhang K., Zheng X. (2018). The chemical compositions of *Angelica pubescens* oil and its prevention of UV-B radiation-induced cutaneous photoaging. Chem. Biodivers..

[7] Kim J., Lee C.W., Kim E.K., Lee S.J., Park N.H., Kim H.S., Kim H.K., Char K., Jang Y.P., Kim J.W. (2011). Inhibition effect of *Gynura procumbens* extract on UV-B-induced matrix-metalloproteinase expression in human dermal fibroblasts. J. Ethnopharmacol..

[8] Parrado C., Mascaraque M., Gilaberte Y., Juarranz A., Gonzalez S. (2016). Fernblock (*Polypodium leucotomos* Extract): Molecular mechanisms and pleiotropic effects in light-related skin conditions, photoaging and skin cancers, a review. Int. J. Mol. Sci..

[9] Bravo K., Duque L., Ferreres F., Moreno D.A., Osorio E. (2017). *Passiflora tarminiana* fruits reduce UVB-induced photoaging in human skin fibroblasts. J. Photochem. Photobiol. B.

[10] Liu S., You L., Zhao Y., Chang X. (2018). Hawthorn polyphenol extract inhibits UVB-induced skin photoaging by regulating MMP expression and type I procollagen production in mice. J. Agric. Food Chem..

[11] Perez-Sanchez A., Barrajo’n-Catala’n E., Caturla N. (2014). Protective effects of citrus and rosemary extracts on UV induced damage in skin cell model and human volunteers. J. Photochem. Photobiol. B Biol..

[12] Noh D., Choi J.G., Lee Y.B., Jang Y.P., Oh M.S. (2019). Protective effects of *Belamcandae rhizoma* against skin damage by ameliorating ultraviolet-B-induced apoptosis and collagen degradation in keratinocytes. Environ. Toxicol..

[13] Rusu M.E., Gheldiu A.M., Mocan A., Vlase L., Popa D.S. (2018). Anti-aging potential of tree nuts with a focus on the phytochemical composition, molecular mechanisms and thermal stability of major bioactive compounds. Food Funct..

[14] Cuelho C.H.F., Alves G.A.D., Lovatto M.O., Bonilha I.F., Barbisan F., da Cruz I.B.M., Oliveira S.M., Fachinetto R., do Canto G.S., Manfron M.P. (2018). Topical formulation containing *Ilex Paraguariensis* extract increases metalloproteinases and myeloperoxidase activities in mice exposed to UVB radiation. J. Photochem. Photobiol. B Biol..

[15] Yang W.S., Lee B.H., Kim S.H., Kim H.G., Yi Y.S., Htwe K.M., Kim Y.D., Yoon K.D., Hong S., Lee W.S. (2013). *Dipterocarpus tuberculatus* ethanol extract strongly suppresses in vitro macrophage-mediated inflammatory responses and in vivo acute gastritis. J. Ethnopharmacol..

[16] Hassan S.M., Al Aqil A.A., Attimarad M. (2013). Determination of crude saponin and total flavonoids content in guar meal. Adv. Med. Plant Res..

[17] Singleton V.L., Joseph A.R. (1965). Colorimetry of total phenolics with phosphomolybdic-phosphotungstic acid reagents. Am. J. Enol. Vitic..

[18] Jia Z., Tang M., Wu J. (1999). The determination of flavonoid contents in mulberry and their scavenging effects on superoxide radicals. Food Chem..

[19] Price M.L., Hagerman A.E., Butler L.G. (1980). Tannin content of cowpeas, chickpeas, pigeon peas, and mung beans. J. Agric. Food Chem..

[20] Bissett D.L., Hannon D.P., Orr T.V. (1987). An animal model of solar-aged skin: Histological, physical, and visible changes in UV-irradiated hairless mouse skin. Photochem. Photobiol..

[21] Lee M.R., Lee G.W., Kim J.E., Yun W.B., Choi J.Y., Park J.J., Kim H.R., Song B.R., Park J.W., Kang M.J. (2019). Biocompatibility of a PLA-based composite containing hydroxyapatite derived from waste bones of dolphin *Neophocaena asiaeorientalis*. J. Aust. Ceram. Soc..

[22] Maria C., Birgit W., Giorgia B., Carla V.C.G., Hermann S., Pidder J.D. (2017). Plant extracts and natural compounds used against UVB-induced photoaging. Biogerontology.

[23] Gruber J.V., Holtz R. (2019). In vitro expression of NLRP inflammasome-induced active Caspase-1 expression in normal human epidermal keratinocytes (NHEK) by various exogenous threats and subsequent inhibition by naturally derived ingredient blends. J. Inflamm. Res..

[24] Hasegawa T., Nakashima M., Suzuki Y. (2016). Nuclear DNA damage-triggered NLRP3 inflammasome activation promotes UVB-induced inflammatory responses in human keratinocytes. Biochem. Biophys. Res. Commun..

[25] Wang S., Zhong J., Li L. (2019). Protective effect of skin-derived precursors on photoaging in nude mice. Australas. J. Dermatol..

[26] Xiao J., Liu B., Zhuang Y. (2019). Effects of rambutan (*Nephelium lappaceum*) peel phenolics and Leu-Ser-Gly-Tyr-Gly-Pro on hairless mice skin photoaging induced by ultraviolet irradiation. Food Chem. Toxicol..

[27] Zhou R., Wang M., Zhang X., Chen A., Fei Y., Zhao Q., Guo D., Chen H., Zheng S. (2020). Therapeutic effect of concentrated growth factor preparation on skin photoaging in a mouse model. J. Int. Med. Res..

[28] Moloney S.J., Learn D.B. (1992). The effect of systemic cyclosporin A on a hairless mouse model of photoaging. Photochem. Photobiol..

[29] McArdle F., Rhodes L.E., Parslew R., Jack C.I.A., Friedmann P.S., Jackson M.J. (2002). UVR-induced oxidative stress in human skin *in vivo*: Effects of oral vitamin C supplementation. Free Radic. Biol. Med..

[30] Ruža P., Borut P., Aleksandar G., Raja D. (2013). Skin photoaging and the role of antioxidants in its prevention. ISRN Dermatol..

[31] Aslam M.S., Ahmad M.S., Mamat A.S. (2015). A phytochemical, ethnomedicinal and pharmacological review of genus *Dipterocarpus*. Int. J. Pharm. Pharm. Sci..

[32] De Abreu H.A., Lago I.A.S., Souza G.P., Piló-Veloso D., Duarte H.A., Alcântara A.F.C. (2008). Antioxidant activity of (+)-bergenin a phytoconstituent isolated from the bark of *Sacoglottis uchi* Huber (Humireaceae). Org. Biomol. Chem..

[33] Zghonda N., Yoshida S., Ezaki S., Otake Y., Murakami C., Mliki A., Ghorbel A., Miyazaki H. (2012). ε-Viniferin is more effective than its monomer resveratrol in improving the functions of vascular endothelial cells and the heart. Biosci. Biotechnol. Biochem..

[34] Yin M.C., Chan K.C. (2007). Nonenzymatic antioxidative and antiglycative effects of oleanolic acid and ursolic acid. J. Agric. Food Chem..

[35] Saleem M., Kim H.J., Jin C., Lee Y.S. (2004). Antioxidant caffeic acid derivatives from leaves of *Parthenocissus tricuspidata*. Arch. Pharm. Res..

[36] Kim D.H., Cho J.Y., Lee I.H., Jin Y.Y., Kang H.S. (2017). Exercise attenuates high-fat diet-induced disease progression in 3xTg-AD mice. Med. Sci. Sports Exerc..

[37] Sun Z., Park S.Y., Hwang E. (2016). Dietary *Foeniculum vulgare* Mill extract attenuated UVB irradiation-induced skin photoaging by activating of Nrf2 and inhibiting MAPK pathways. Phytomedicine.

[38] Lim J.Y., Kim O.K., Lee J.M., Lee M.J., Kang N.I., Hwang J.K. (2014). Protective effect of the standardized green tea seed extract on UVB-induced skin photoaging in hairless mice. Nutr. Res. Pract..

[39] Suzuki T., Yamamoto M. (2015). Molecular basis of the Keap1-Nrf2 system. Free Radic. Biol. Med..

[40] Ishii T., Itoh K., Yamamoto M. (2002). Roles of Nrf2 in activation of antioxidant enzyme genes via antioxidant responsive elements. Methods Enzymol..

[41] Wu P.Y., Lyu J.L., Liu Y.J., Chien T.Y., Hsu H.C., Wen K.C., Chiang H.M. (2017). Fisetin regulates Nrf2 expression and the inflammation-related signaling pathway to prevent UVB-induced skin damage in hairless mice. Int. J. Mol. Sci..

[42] Xian D., Gao X., Xiong X., Xu J., Yang L., Pan L., Zhong J. (2017). Photoprotection against UV-induced damage by skin-derived precursors in hairless mice. J. Photochem. Photobiol. B.

[43] Durchdewald M., Beyer T.A., Johnson D.A., Johnson J., Werner S., auf dem Keller U. (2007). Electrophilic chemicals but not UV irradiation or reactive oxygen species activate Nrf2 in keratinocytes in vitro and in vivo. J. Investig. Dermatol..

[44] Choi S.Y., Hong J.Y., Ko E.J., Kim B.J., Hong S.W., Lim M.H., Yeon S.H., Son R.H. (2018). Protective effects of fermented honeybush (*Cyclopia intermedia*) extract (HU-018) against skin aging: A randomized, double-blinded, placebo-controlled study. J. Cosmet. Laser Ther..

[45] Yun M.S., Kim C., Hwang J.K. (2019). *Agastache rugosa* Kuntze attenuates UVB-induced photoaging in hairless mice through the regulation of MAPK/AP-1 and TGF-β/ Smad pathways. J. Microbiol. Biotechnol..

[46] Im A.R., Yeon S.H., Ji K.Y., Son R.H., Um K.A., Chae S. (2020). Skin hydration effects of scale-up fermented *Cyclopia intermedia* against ultraviolet B-induced damage in keratinocyte cells and hairless mice. Evid. Based Complement. Alternat. Med..

[47] Yanagihara S., Kobayashi H., Tamiya H., Tsuruta D., Okano Y., Takahashi K., Masaki H., Yamada T., Hasegawa S., Akamatsu H. (2013). Protective effect of hochuekkito, a Kampo prescription, against ultraviolet B irradiation-induced skin damage in hairless mice. J. Dermatol..

[48] Aguirre-Cruz G., León-López A., Cruz-Gómez V., Jiménez-Alvarado R., Aguirre-Álvarez G. (2020). Collagen hydrolysates for skin protection: Oral administration and topical formulation. Antioxidants.

[49] Zattra E., Coleman C., Arad S., Helms E., Levine D., Bord E., Guillaume A., El-Hajahmad M., Zwart E., van Steeg H. (2009). *Polypodium leucotomos* extract decreases UV-induced Cox-2 expression and inflammation, enhances DNA repair, and decreases mutagenesis in hairless mice. Am. J. Pathol..

[50] Alcaraz M.V., Pathak M.A., Rius F., Kollias N., González S.V. (1999). An extract of *Polypodium leucotomos* appears to minimize certain photoaging changes in a hairless albino mouse animal model. A pilot study. Photodermatol. Photoimmunol. Photomed..

[51] Vayalil P.K., Elmets C.A., Katiyar S.K. (2003). Treatment of green tea polyphenols in hydrophilic cream prevents UVB-induced oxidation of lipids and proteins, depletion of antioxidant enzymes and phosphorylation of MAPK proteins in SKH-1 hairless mouse skin. Mol. Carcinog..

[52] Hwang E., Sun Z.W., Lee T.H., Shin H.S., Park S.Y., Lee D.G., Cho B.G., Sohn H., Kwon O.W., Kim S.Y. (2013). Enzyme-processed Korean Red Ginseng extracts protects against skin damage induced by UVB irradiation in hairless mice. J. Ginseng. Res..

[53] Hwang E., Lee T.H., Park S.Y., Yi T.H., Kim S.Y. (2014). Enzyme-modified Panax ginseng inhibits UVB-induced skin aging through the regulation of procollagen type I and MMP-1 expression. Food Funct..

[54] Magcwebeba T., Swart P., Swanevelder S. (2016). Antiinflammatory effects of *Aspalathus linearis* and *Cyclopia* spp. extracts in a UVB/keratinocyte (HaCaT) model utilising interleukin-1α accumulation as biomarker. Molecules.

[55] Nistico S., Ehrlich J., Gliozzi M. (2015). Telomere and telomerase modulation by bergamot polyphenolic fraction in experimental photoageing in human keratinocytes. J. Biol. Regul. Homeost. Agents.

[56] Lee T.H., Do M.H., Oh Y.L. (2014). Dietary fermented soybean suppresses UVB-induced skin inflammation in hairless mice via regulation of the MAPK signaling pathway. J. Agric. Food Chem..

[57] Wang Z., Zhang S., Xiao Y., Zhang W., Wu S., Qin T., Yue Y., Qian W., Li L. (2020). NLRP3 inflammasome and inflammatory diseases. Oxid. Med. Cell Longev..

[58] Schroder K., Tschopp J. (2010). The inflammasomes. Cell.

[59] Jo E.K., Kim J.K., Shin D.M., Sasakawa C. (2016). Molecular mechanisms regulating NLRP3 inflammasome activation. Cell Mol. Immunol..

[60] Boyden E.D., Dietrich W.F. (2006). Nalp1b controls mouse macrophage susceptibility to anthrax lethal toxin. Nat. Genet..

[61] Miao E.A., Alpuche-Aranda C.M., Dors M., Clark A.E., Bader M.W., Miller S.I., Aderem A. (2006). Cytoplasmic flagellin activates caspase-1 and secretion of interleukin 1beta via Ipaf. Nat. Immunol..

[62] Zhao Y., Yang J., Shi J., Gong Y.N., Lu Q., Xu H., Liu L., Shao F. (2011). The NLRC4 inflammasome receptors for bacterial flagellin and type III secretion apparatus. Nature.

[63] Martinon F., Petrilli V., Mayor A., Tardivel A., Tschopp J. (2006). Gout-associated uric acid crystals activate the NALP3 inflammasome. Nature.

[64] Fernandes-Alnemri T., Yu J.W., Datta P., Wu J., Alnemri E.S. (2009). AIM2 activates the inflammasome and cell death in response to cytoplasmic DNA. Nature.

[65] Rodríguez-Luna A., Ávila-Román J., Oliveira H., Motilva V., Talero E. (2019). Fucoxanthin and rosmarinic acid combination has anti-inflammatory effects through regulation of NLRP3 inflammasome in UVB-exposed HaCaT keratinocytes. Mar. Drugs..

