# Supplementary material for: Therapeutic Effects of Dipterocarpus tuberculatus with High Antioxidative Activity Against UV-Induced Photoaging of NHDF Cells and Nude Mice
Source: Antioxidants (Basel). 2021 May 17;10(5):791. doi: 10.3390/antiox10050791 (PMC8157063; doi:10.3390/antiox10050791)
Supplement: Supplementary file 1 [file antioxidants-10-00791-s001.zip › antioxidants-1200281-supplementary.pdf]

Supplementary Materials

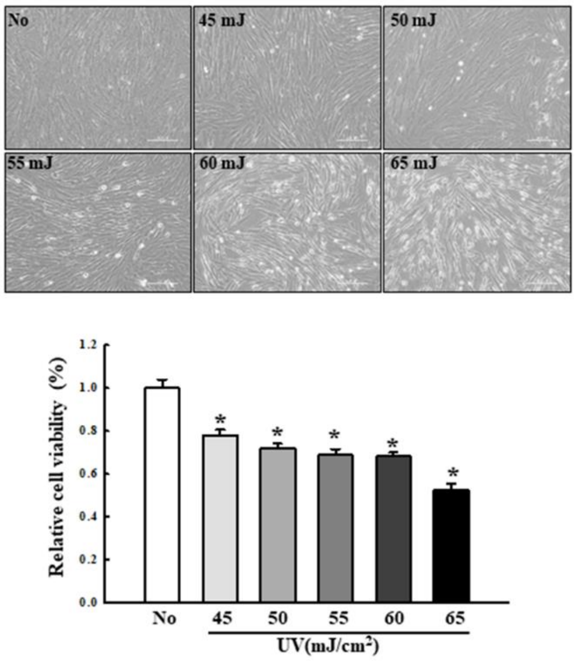

Figure S1. Determination of optimal UV dosage. NHDF cells were exposed with five different doses of UVB (45, 50, 55, 60 and 65 mJ/cm^2), and the morphological changes of cells were observed under a microscope at 400× magnification. The viability of these cells was determined with MTT assay. Two to three wells per group were used in the MTT assay, and optical density was measured in duplicates. Data are reported as the means ± SD. *, $p<0.05$ relative to the No treated group.

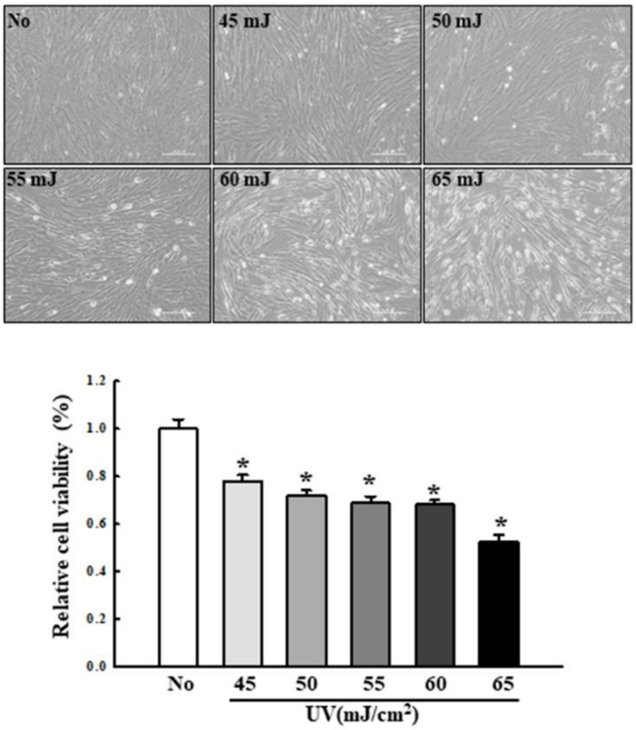

Figure S2. Cytotoxicity of MED. NHDF cells were exposed with three different doses of MED (100, 200 and 400 μg/mL) of MED for 24 h, and the morphological changes of cells were observed under a microscope at 400× magnification. The viability of these cells was determined with MTT assay. Two to three wells per group were used in the MTT assay, and optical density was measured in duplicates. Data are reported as the means ± SD.

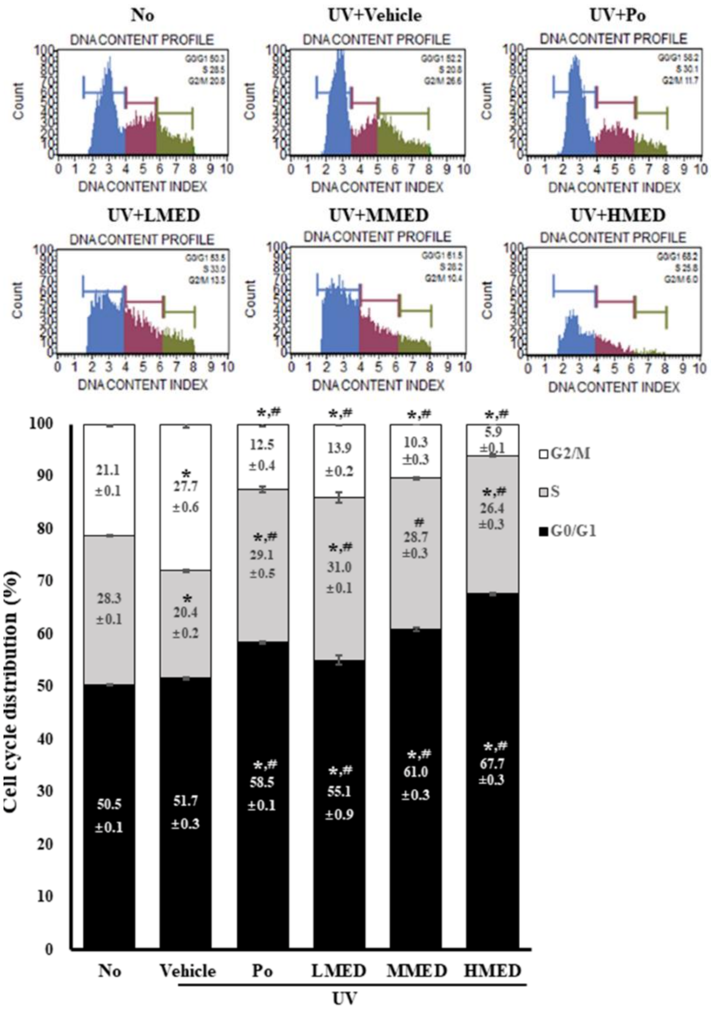

Figure S3. Cell cycle analysis of MED treated NHDF cells. After treatment with 100, 200 and 400 µg/mL MED for 24 h, the number of NHDF cells in the G0/G1, S and G2/M stages were analyzed. Two to three wells per group were used for PI staining, and the cell number in each phase was measured in duplicate. Data are reported as the mean ± SD. *, $p<0.05$ compared to the No treated group. #, $p<0.05$ compared to the UV+Vehicle treated group.

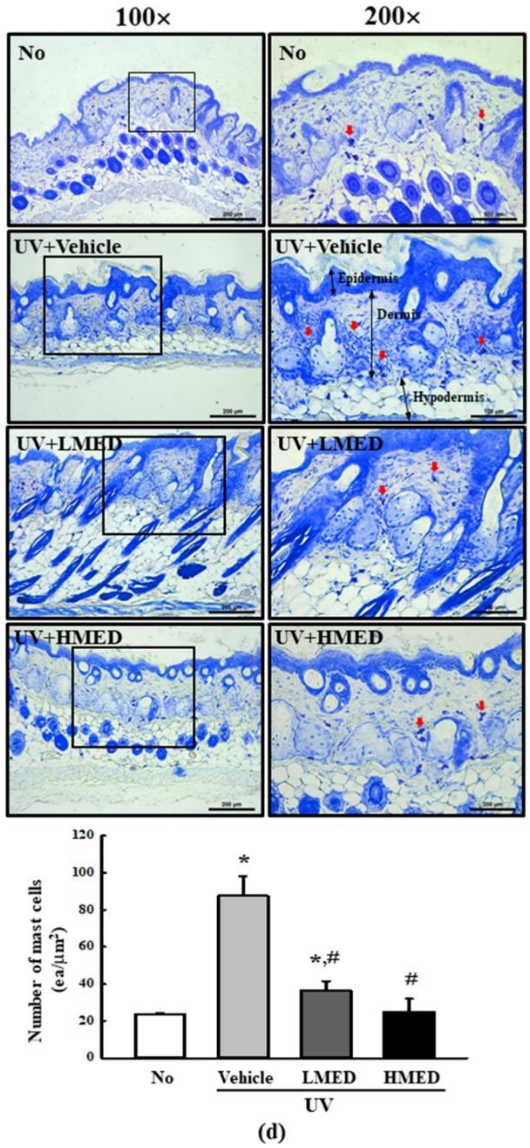

Figure S4. Infiltration of mast cells. Infiltration of mast cells was identified in the slide sections of ear tissue stained with toluidine blue, followed by observation at 400× magnification. Arrows indicate the infiltrated mast cells in the dermis of the ear tissue. Three to four mice per group were used in the preparation of tissue slides. Mast cell counting were analyzed in duplicate for each sample.

Table S1. Antibodies list for Western blot analyses.

Name	Cat. No.	Company
Anti-Bax	Ab7977	Abcam, Cambridge, UK
Anti-Bcl-2	Ab7973	Abcam, Cambridge, UK
Anti-Cas-3	9662	Cell Signaling, Danvers, MA, USA
Anti-actin	4967	Cell Signaling, Danvers, MA, USA
Anti-Nrf2	Ab137550	Abcam, Cambridge, UK
Anti-SOD	Ab80946	Abcam, Cambridge, UK
Anti-ASC	67824	Cell Signaling, Danvers, MA, USA
Anti-Cas-1	24232	Cell Signaling, Danvers, MA, USA
Anti-NLRP3	15101	Cell Signaling, Danvers, MA, USA
Anti-iNOS	PA3-030A	Thermo Fisher Scientific, MA, USA
Anti-Cox-2	4842	Cell Signaling, Danvers, MA, USA
Anti-MMP-9	SC-13520	Santa Cruz Biotechnology, CA, USA
Anti-MMP-2	87809	Cell Signaling, Danvers, MA, USA

Table S2. Primer sequence for RT-PCR.

Primer name	Sequence (from 5' to 3')	Product size (bp)
TNF-α		
Forward	CCT GTA GCC CAC GTC GTA GC	374
Reverse	TTG ACC TCA GCG CTG ACT TG	
IL-6		
Forward	TTG GGA CTG ATG TTG TTG ACA	200
Reverse	TCA TCG CTG TTG ATA CAA TCA GA	
IL-1β		
Forward	CAG TTC TGC CAT TGA CCA T	218
Reverse	TCT CAC TGA A AC TCA GCC GT	
NF-κB		
Forward	GTA ACA GCA GGA CCC AAG GA	501
Reverse	AGC CCC TAA TAC ACG CCT CT	
β-actin		
Forward	TGG AAT CCT GTG GCA TCC ATG AAA C	349
Reverse	TAA AAC GCA GCT CAG TAA CAG TCC G	
